# Systemic Associations with Keratoconus

**DOI:** 10.3390/life13061363

**Published:** 2023-06-10

**Authors:** Prasida Unni, Hyunjoo Jean Lee

**Affiliations:** 1 Chobanian and Avedisian School of Medicine, Boston University, Boston, MA 02118, USA; punni@bu.edu; 2 Department of Ophthalmology, Boston Medical Center, Boston, MA 02118, USA

**Keywords:** keratoconus, associations, atopy

## Abstract

Keratoconus is a disease of the cornea that results in progressive steepening and thinning of the cornea and subsequent vision loss. It nearly always presents as a bilateral disease, suggesting that there is an underlying abnormality of the corneas that becomes manifest with time. However, the mechanisms underlying the development of keratoconus are largely unknown. Associations reported between keratoconus and systemic diseases are abundant in the literature, and the list of possible associations is very long. We found that atopy, Down syndrome, and various connective tissue diseases were the most frequently cited associations in our broad literature search. Additionally, Diabetes Mellitus has been increasingly studied as a possible protective factor against keratoconus. In this review, we have summarized the evidence for and against these particular systemic conditions and keratoconus and have discussed some of the implications of keratoconus patients having these conditions.

## 1. Introduction

Keratoconus is a progressive disease that results in what is known as ectasia, which is a steepening and thinning of the cornea. Patients experience vision loss due to progressive irregular astigmatism, typically in both eyes, although the condition can be very asymmetric. The prevalence of keratoconus is estimated to be between 1 in 375 to 1 in 2000 individuals and typically presents in the second decade of life [1,2,3]. How and why keratoconus develops is still largely unknown, but genetic factors and heredity are known to play a role [4]. Additionally, there have been multiple associations made between keratoconus and various systemic conditions, and these associations could serve as clues to the pathogenesis of keratoconus. Some of these systemic associations have been corroborated by multiple controlled studies, while others are only suggested by small observational studies. In this review, we aimed to summarize the evidence for the most frequent systemic associations of keratoconus.

## 2. Methods

We searched the PubMed (U.S. National Library of Medicine) database using a combination of the following search terms: (“systemic association” OR co-morbid* OR associat*) AND (“keratoconus” [Mesh] OR “keratoconus”). This resulted in 1494 results, of which 138 articles were review articles. Table 1 lists the conditions we found reported to be associated with keratoconus.

We reviewed the titles and abstracts of the review articles and identified the systemic conditions most frequently associated with keratoconus. Of the identified systemic associations of keratoconus, atopy and Down syndrome were discussed most frequently. Several other associations, including Ehlers–Danlos syndrome, Marfan syndrome, Osteogenesis Imperfecta, and mitral valve prolapse, which all fall under the umbrella of connective tissue diseases, were also represented frequently in the literature search results. Finally, the association between keratoconus and diabetes was of particular interest given the inverse relationship between rates of keratoconus and diabetes reported in several studies, suggesting a potential protective effect of diabetes. Thus, for the purposes of this review, we selected atopy, Down syndrome, connective tissue diseases, and diabetes as associations of interest.

Articles for each of these associations were gathered through specific PubMed search terms. The search term (“keratoconus”) AND (“allerg*” OR “atop*”) yielded 348 results for atopic associations with keratoconus. The search term (“keratoconus”) AND (“down syndrome”) yielded 128 results for associations between Down syndrome and keratoconus. The search terms (“keratoconus”) AND (“ehlers-danlos”), (“keratoconus”) AND (“marfan”), (“keratoconus”) AND (“osteogenesis imperfecta”), and (“keratoconus”) AND (“mitral valve prolapse”) yielded 31, 25, 17, and 21 results, respectively. Finally, the search term (“keratoconus”) AND (“diabetes”) yielded 110 results for associations between diabetes and keratoconus. The results gathered from these search terms were reviewed to delineate the nature and strength of each association with keratoconus.

## 3. Results

### 3.1. Keratoconus and Atopy

#### 3.1.1. Inflammation and Allergy-Related Markers in Keratoconus

Atopy refers to a predisposition to allergen hypersensitivity mediated by CD4+ Th2 differentiation and overproduction of immunoglobulin E (IgE) [5]. Several diseases fall within the classification of atopy, notably asthma, atopic dermatitis (eczema), allergic rhinitis, and ocular allergy (allergic conjunctivitis and vernal keratoconjunctivitis).

There is evidence that allergy-related processes play a role in the pathogenesis of keratoconus. Keratoconus patients have a significantly higher incidence of elevated serum IgE (52%) compared to controls (7%) [6]. Several other inflammatory molecules, including MMP-1, MMP-3, MMP-7, MMP-13, IL-4, IL-5, IL-6, IL-8, TNFa, and TNFb, have been found to be elevated in tear samples from keratoconus patients [7]. Eye-rubbing behavior, which is associated with atopic disease, also results in increases in MMP-13, IL-6, and TNFa in normal subject tears [8]. IL-6 has also been found to be correlated with keratoconus severity, with increasing IL-6 concentrations in tear samples associated with more severe keratoconus [9].

While atopy and keratoconus share pertinent molecular markers, it is still largely unclear whether atopy acts directly or indirectly, via behaviors such as eye rubbing, to serve as a risk factor for keratoconus. An examination of two allergy-related single nucleotide polymorphisms (SNPs), IL4 rs2070874 and FOXP3 rs3761548, found no significant difference in allele and genotype frequencies between keratoconus and control groups, despite there being a significantly higher rate of atopy in the keratoconus group [10]. Additionally, keratoconus patients with atopic dermatitis were found to have a lower-than-expected frequency of filaggrin (FLG) mutations, despite FLG mutations being a strong genetic risk factor for atopic dermatitis [11]. However, the absence of these genetic associations by no means rules out the possibility that atopy plays a direct role in the pathogenesis of keratoconus.

#### 3.1.2. Prevalence of Atopy within Keratoconus Populations

Multiple case–control studies have found an association between atopic disease and keratoconus (Table 2). Only a few studies have found no association between atopy and keratoconus. One of these studies was a case–control study that did not find an increased rate of atopy in a keratoconus population, but this study was limited by a small sample size of 30 keratoconus patients and 30 age- and sex-matched control participants [12]. Another study analyzed keratoconus associations using the Korean National Health Insurance Service National Sample Cohort database and did not find an association between keratoconus and atopy diagnoses [13]. However, the authors did find a significantly higher rate of allergy-related diseases, such as allergic conjunctivitis, in the keratoconus population. The third study we found that did not find a difference in rates of atopy between keratoconus and control populations was primarily aimed at comparing ocular surface Toll-like receptor expression between controls, keratoconus, and first-degree relatives of keratoconus patients and only examined rates of atopy in the subjects in order to characterize their population; the sample size was also relatively small (36 controls, 60 keratoconus) [14]. Although not statistically significant, they did report that the rate of “allergic disease” in keratoconus patients was 60%, compared to 47.2% in controls. The limitations of each of these studies may account for why no association between atopy and keratoconus was found, whereas the majority of studies have found significantly higher rates of atopy in keratoconus patients compared to controls [9,10,15,16,17,18,19].

Additionally, studies have found significantly higher rates of asthma [20,21,22,23,24,25,26,27], allergic rhinitis [20,21,23,24,25,27], atopic dermatitis [24,25], and ocular allergy [13,22,25] in keratoconus populations (Supplemental Tables S1–S4). A multivariate regression analysis found that asthma was associated with increased odds of keratoconus diagnosis, although, on the contrary, allergic rhinitis was associated with reduced odds of severe KCN compared to mild KCN [27]. One study found that vernal keratoconjunctivitis was associated with more severe forms of keratoconus in a younger patient population [28]. Another study found that allergy was associated with earlier onset of keratoconus [29].

Environmental pollution has recently been implicated in the development of keratoconus, where a moderate to strong correlation was found between keratoconus prevalence and levels of fine particulate matter in the air [30]. However, whether this effect is due to exacerbating manifestations of atopy, increased eye rubbing, or both has yet to be determined.

#### 3.1.3. Atopy or Eye Rubbing?

An important point of debate regarding whether atopy is a risk factor for keratoconus relates to whether it is the atopic disease or behaviors related to the atopic disease that serves as the primary risk factor for keratoconus. Eye rubbing is a behavior that is commonly seen in patients with atopic disease. Several studies have found a significant association between eye rubbing and keratoconus. In one multivariate analysis, eye rubbing was the only significant predictor of keratoconus, whereas atopy was only significant in their univariate analysis [16]. Other studies have found both eye rubbing and atopy to be independently associated with keratoconus, and the combination of the two risk factors greatly increases the odds of having keratoconus [19]. Sleep position, pertaining to contact between the hand or forearm of patients with their eyes, has also been found to be significantly associated with keratoconus [22]. Additionally, an increased risk of keratoplasty, a marker of severe disease status, has also been observed in keratoconus patients exhibiting eye-rubbing behavior [31].

As mentioned above, eye rubbing can result in the release of pro-inflammatory cytokines and thus could be contributing to keratoconus development indirectly through mechanisms related to atopy and inflammation [8]. On the other hand, eye rubbing has been proposed to also cause mechanical and thermal stress to the cornea [32]. Given the high prevalence of eye rubbing in patients with ocular allergy and atopy, it is still not entirely clear whether eye rubbing has a causative role in keratoconus, despite some independent associations found between eye rubbing and keratoconus. We were not able to find further studies addressing whether eye rubbing directly contributes to keratoconus progression in atopic patients. Studies investigating keratoconus progression in patients who successfully stop eye rubbing could help determine the importance of eye rubbing to the progression of keratoconus.

#### 3.1.4. Complications and Progression of Keratoconus in Atopic Patients

Corneal hydrops is a complication of keratoconus that occurs when aqueous fluid enters the stroma via a rupture in Descemet’s layer, leading to severe vision impairment. A higher risk for developing corneal hydrops has been observed in keratoconus patients with earlier onset disease, eye rubbing behavior, and atopy [31]. Further, a multivariate analysis has demonstrated independent associations of vernal keratoconjunctivitis and asthma with odds of hydrops in keratoconus patients [33]. Eye rubbing has also been associated with the development of acute corneal hydrops in keratoconus patients [34].

Despite the significant association between atopy and keratoconus supported by much of the literature, the effect of atopy on the progression of keratoconus is unclear. An early study on this topic, examining demographic and systemic associations with rates of progressing to penetrating keratoplasty prior to the advent of collagen cross-linking, found no influence of atopy on keratoconus progression [35]. A more recent study of 96 eyes of 96 patients with a 39.6% progression rate also found that atopy was not a significant risk factor for progression detected by tomography over the course of their study [36]. On the other hand, severe atopic dermatitis has been associated with an increased risk for progression to corneal transplantation [37]. There have also been studies examining risk factors for progression despite corneal cross-linking that have suggested a link between atopy and failure of cross-linking to completely halt disease progression. In a retrospective study of eyes demonstrating progression despite corneal cross-linking, 75% of the 20 eyes of 20 patients with progression had allergic conjunctivitis [38]. In a separate study, 5 of 5 patients who progressed after corneal cross-linking in one or both eyes all reported a history of allergic conjunctivitis and eye rubbing [39]. However, there were no comparisons made between rates of allergic conjunctivitis, atopy, and eye rubbing between the progressive and non-progressive groups in either cross-linking study, making any actual associations unclear.

There is some mixed evidence that corneal transplantation complications could be greater in keratoconus patients with atopy. Patients with ocular allergy and longer duration of corneal hydrops were found to have a higher risk of endothelial rejection, although long-term allograft survival and vision outcomes remained strong [40]. Other studies have not found higher rates of graft rejection in patients with atopy [41] nor any significance in graft survival after the first, second, third, or further grafts [42]. Further, keratoconus patients with and without vernal keratoconjunctivitis have been found to have similar rates of graft survival, postoperative complications, and visual results after penetrating keratoplasty [43]. On the other hand, a retrospective study found that 17% of keratoconus patients who had atopic dermatitis developed post-keratoplasty atopic sclerokeratitis (PKAS) [44].

### 3.2. Keratoconus and Down Syndrome

#### 3.2.1. Prevalence of Down Syndrome within Keratoconus Populations

Down syndrome is a genomic disorder caused by trisomy of chromosome 21 (HSA21), which is known to be the most common genomic disorder of intellectual disability [45]. Down syndrome is associated with many systemic manifestations and co-existing medical conditions, including congenital heart disease, pulmonary hypertension, hearing deficits, hematologic and oncological disorders, sleep disorders, thyroid abnormalities, dysphagia, seizure disorders, gastrointestinal anomalies, and visual problems [46]. Among these visual problems, refractive error, amblyopia, strabismus, nystagmus, nasolacrimal duct obstruction, and keratoconus are all ophthalmic manifestations associated with Down syndrome [47].

The prevalence of keratoconus in Down syndrome patients has been reported as ranging from 0% to 71% across the literature [48], with the variation being possibly attributed to patients’ age, ethnicity, sample size of the study, and the variety of diagnostic criteria and technologies used [49]. Several studies examining the prevalence of ocular disease in children from various ethnic populations did not detect keratoconus in their Down syndrome populations [50,51,52,53]. However, none of these studies used topography or tomography to detect keratoconus, and they included very young subjects, including infants and very few older children. Keratoconus does not typically manifest until the second decade of life, and therefore, the prevalence of keratoconus in these studied populations would be expected to be very low at most. In contrast, a review of keratoconus prevalence rates in Down syndrome patients found higher rates (>10%) of keratoconus reported in studies that utilized corneal topography as the standard diagnostic tool [54]. Furthermore, a more recent study of pediatric patients with Down syndrome detected keratoconus suspicion or definite keratoconus using Schiempflug tomography in at least one eye of 32% of patients [55], again suggesting that with more sensitive diagnostic technologies, keratoconus is more likely to be detected.

Unlike the many case–control studies that examine the rate of atopy in keratoconus and control populations, few such studies exist for determining the rate of Down syndrome in keratoconus and control groups. In a multi-center observation study of 217 patients, Down syndrome patients were observed to have steeper, thinner corneas and more corneal aberrations compared to non-Down syndrome controls, with topography findings often suggestive of keratoconus [49]. A population-based study of over 5000 patients examining risk factors contributing to keratoconus in Taiwan found a significantly higher prevalence of Down syndrome in the keratoconus population compared to controls, with rates of 0.38 and 0.05, respectively [23]. Another large-scale study of 1653 keratoconus patients matched 1:1 with subjects without keratoconus also reported a significantly higher rate of Down syndrome in keratoconus patients compared to controls, with keratoconus patients having a six-fold greater odds of Down syndrome [27]. An earlier multivariate analysis found no significant difference in Down syndrome rates between keratoconus and control groups [16], but this study was much smaller (only 120 subjects) compared to the two aforementioned studies.

#### 3.2.2. Eye Rubbing in Down Syndrome

As discussed earlier in the context of atopy, eye rubbing has been identified as a risk factor for the pathogenesis and progression of keratoconus. While several studies have suggested that Down syndrome patients may be at higher risk of developing keratoconus due to eye-rubbing behavior [56,57,58], the evidence to support this association is limited. One study examining parental subjective assessment of eye rubbing in their child with Down syndrome found no significant association between reported eye rubbing frequency and corneal topographic abnormalities [59]. Another study investigating the prevalence of keratoconus in 250 Down syndrome patients found no significant difference in the rate of eye rubbing behavior between patients with and without keratoconus or between keratoconus patients with non-progressive and progressive disease [60]. The connection between eye rubbing and keratoconus remains a topic requiring further investigation, particularly in the context of eye rubbing rates in Down syndrome patients and whether this behavior increases the risk of Down syndrome patients developing keratoconus.

#### 3.2.3. Caring for Down Syndrome Patients with Keratoconus

Diagnosing and treating keratoconus at the early stages of its manifestation is critical for slowing the progression of the disease and preserving sight. Given the evidence for a higher prevalence of keratoconus in Down syndrome patients, along with the constellation of ocular abnormalities associated with Down syndrome, it is important for all Down syndrome patients to be regularly screened for corneal changes. An assessment of tomographic indices for keratoconus diagnosis in Down syndrome patients suggested that anterior higher-order aberrations, posterior vertical coma, anterior vertical coma, and total higher-order aberrations were effective keratoconus discriminators in this population [61].

Early detection and treatment of keratoconus also provide opportunities for earlier interventional procedures, such as corneal cross-linking. Subsequently, this can deter a patient’s need for future penetrating keratoplasty, which has been shown to prove more challenging for Down syndrome patients in regard to postoperative care [62].

### 3.3. Keratoconus and Diseases of Connective Tissue

#### 3.3.1. Ehlers–Danlos Syndrome

Ehlers–Danlos Syndrome (EDS) is characterized by clinical manifestations of skin hyperextensibility, joint hypermobility, and tissue fragility [63]. The various subtypes of EDS have been found in association with 19 genes related to collagen and extracellular matrix (ECM) synthesis [64]. A 1975 study of joint hypermobility in 44 keratoconus patients found 50% of patients to have some degree of hypermobility in their joints, although none of these patients fit the full criteria for EDS diagnosis, which includes skin hyperextensibility and fragility, as well as a severe level of joint hypermobility [65]. An association between keratoconus and EDS is supported by findings that keratoconus patients are five times more likely to demonstrate hypermobility of the metacarpophalangeal and wrist joints [66]. However, there are few studies that have explored the prevalence of EDS in keratoconus patients at a larger scale, with the aforementioned studies having only had less than 100 subjects.

Data regarding the findings of keratoconus in patients with EDS is mixed. Small-scale studies have noted keratoconus diagnoses in patients with EDS spondylodysplastic form Type III [67] and in patients with EDS Type VI [68]. However, an examination of 36 EDS patients found no keratoconus via slit lamp biomicroscopy, retinoscopy, and videokeratography methods [69]. Another study of patients with EDS kyphoscoliotic form Type VI noted corneal abnormalities in the form of limbus-to-limbus corneal thinning but no keratoconus [70].

Although there is weak evidence for a clear clinical association between keratoconus and EDS, several studies have examined genetic mediators that keratoconus and EDS could have in common. A recent study utilized whole exome sequencing (WES) to identify 34 keratoconus-related genes and noted genetic variation for several genes that are pertinent to EDS, including COL5A1, TNXB, ZNF469, and COL12A1 [71]. These results support previous findings of sequence variants in COL5A1 that could contribute to reduced central corneal thickness (CCT) in both keratoconus and EDS [72]. Homozygous mutations in ZNF469, another gene related to EDS, result in brittle cornea syndrome (BCS), which is characterized by extreme corneal thinning. ZNF469 alleles with low predicted pathogenicity were found to be enriched in 12.5% of keratoconus patients and could be a significant genetic risk factor for keratoconus [73]. While there is some genetic basis for an association between keratoconus and EDS, it appears that EDS does not typically lead to the development of keratoconus, and the EDS-like symptoms observed in some keratoconus patients are too mild to fit the full criteria for EDS diagnosis.

#### 3.3.2. Marfan Syndrome

Marfan Syndrome (MFS) is an autosomal dominant disorder with variable penetrance, characterized by musculoskeletal manifestations of exaggerated long bone growth, accompanied by ocular, craniofacial, neurologic, pulmonary, craniofacial, and cardiopulmonary manifestations [74]. Most cases of MFS can be attributed to mutations in the fibrillin-1 gene [75]. While case reports have identified keratoconus in patients with Marfan syndrome [76,77], there is very weak evidence for a significant clinical association between these two diseases. Earlier studies have noted corneal abnormalities in MFS patients, including flattening and astigmatism [78], and an evaluation of 160 MFS patients found no keratoconus in any of these patients [79]. This corresponds with a case–control study of MFS and normal control subjects, which also found no indications of keratoconus in MFS eyes [80]. Further, a case–control study of keratoconus and control participants found no difference in the prevalence of MFS between these groups [16]. Evidently, there is very little support for the association between keratoconus and MFS, despite MFS being widely cited as an association of keratoconus [81,82,83,84,85,86,87,88,89,90,91].

#### 3.3.3. Osteogenesis Imperfecta

Osteogenesis Imperfecta (OI) typically presents as an autosomal dominant disorder linked to mutations in genes essential for type 1 collagen synthesis, and common clinical manifestations include bone fragility, decreased bone mass, blue sclera, fractures, and Wormian bones of the skull [92]. Subtypes of OI, classified as Types 1–4, have been determined based largely on the radiographic variation and clinical presentation of OI patients [93]. Given the abundance of Type I collagen in the corneal stroma [94], a few studies have explored the link between OI and corneal diseases such as keratoconus. An analysis of an Italian family with Type 1 OI found keratoconus present in several family members, with a notably very early presentation of keratoconus in a family member who was only 3 years old at the time of the study [95]. Further, a case–control study of 23 OI and 25 normal control subjects found that OI patients have significantly lower CCT compared to age-, sex-, and refraction-matched controls [96].

As seen with EDS and MFS, case–control studies of patients with OI and keratoconus are scarce. Very recently, a study comparing 23 OI patients, 99 keratoconus patients, and 92 normal control patients, utilizing the Corvis ST (Oculus, Wetzlar, Germany) to identify biomechanical differences, found that OI and keratoconus corneas were less stable and easier to deform compared to control corneas, but only keratoconus corneas demonstrated significantly lower material stiffness [97]. While this study cannot address the question of whether keratoconus is more prevalent in the OI patient population, it does indicate that the biomechanical effects on the cornea of OI and keratoconus are different.

#### 3.3.4. Mitral Valve Prolapse

Mitral valve prolapse (MVP) is a cardiac valvular disorder that has two common mechanisms of manifestations: (1) altered collagen organization and thickening of valve leaflets due to fibromyxomatous changes and (2) collagen accumulation and thickened spongiosa due to fibroelastic deficiency [98]. Among the connective tissue disorders discussed in this review, the association between MVP and keratoconus arguably has the strongest evidence.

Early studies of the association between MVP and keratoconus had mixed results. A case–control cross-sectional study with 95 keratoconus patients and 96 matched controls examined all subjects via M-mode ultrasound, echocardiography, and cardiac auscultation methods and found no significant difference in the prevalence of MVP between these two groups [99]. However, other early studies found significantly higher rates of MVP in keratoconus groups compared to age- and sex-matched controls, with one study citing 38% of keratoconus patients displaying MVP compared to 13% of controls [100] and another demonstrating 58% of keratoconus patients displaying MVP compared to 7% controls [101]. Another study examining 35 MVP patients and 25 controls found a borderline association between MVP and keratoconus, with 11.1% of MVP eyes demonstrating keratoconus compared to only 2% of control eyes [102]. These findings have been supported by a later study using a Scheimpflug camera combined with Placido corneal topographic diagnosis that found a significantly higher rate of keratoconus in MVP patients (13.4%) compared to controls (1.1%) [103]. It also appears that MVP may be more prevalent in populations with more severe cases of keratoconus. A study examining keratoconus patients with complications of corneal hydrops found that this group had a significantly higher prevalence of MVP (65.6%) compared to controls (9%) [104].

The evidence for the association between MVP and keratoconus in large-scale studies is also mixed but perhaps more inclined to support the existence of an association. A population-based matched case–control study in Taiwan did not find a significant difference in rates of MVP between keratoconus and control subject groups [23]. However, a meta-analysis of six studies reporting the prevalence of MVP in keratoconus populations, and two studies reporting the prevalence of keratoconus in the MVP population, found a significant association between MVP and keratoconus [105]. Further, a retrospective nationwide matched-cohort study in Taiwan examining 4488 keratoconus patients proposed recommendations for screening keratoconus patients for MVP after finding a 1.77 times higher incidence of MVP in keratoconus patients 40 years and older and a 1.49 times higher incidence of MVP in female keratoconus patients compared to controls [106].

### 3.4. Keratoconus and Diabetes Mellitus

#### 3.4.1. Corneal Characteristics of Diabetic Patients

Diabetes mellitus (DM) is a condition related to a deficiency in the hormone insulin, as seen in Type 1 DM, or associated with the development of insulin resistance, as seen in Type 2 DM. Diabetic conditions are associated with a wide range of systemic manifestations, including a predisposition to hypertension, arterial stiffness, systemic inflammation, and cardiovascular disease [107]. Central corneal thickness (CCT) is an important measure for evaluating keratoconus, with lower CCT being a major criterion for the diagnosis of keratoconus. Several studies examining CCT in patients with diabetes have found significantly higher CCT values in diabetic patients compared to healthy control subjects, while on the other hand, a few reports have found no significant CCT increase in patients with diabetes [108,109,110,111,112,113]. One study found that the duration of diabetes positively correlates with higher CCT, as seen in patients with diabetes for over 10 years, demonstrating significantly higher CCT compared to those who have had diabetes for less than 10 years [112]. Interestingly, a clinical trial examining CCT in diabetic patients prior to achieving good glycemic control (HbA1C levels above 7%) and after treatment at an endocrinology clinic to lower HbA1C levels below 7% found that CCT measurements decreased significantly in patients after achieving good glycemic control [114]. While keratoconus is characterized by progressive thinning and decreased stiffness of the corneal stroma, DM has been associated with increased corneal rigidity [110,115].

#### 3.4.2. A Possible Protective Effect of Diabetes Mellitus in Keratoconus

Given reports of higher CCT and increased corneal stiffness in DM patients, the potential for DM to provide a protective effect against keratoconus has become a topic of interest. It has been hypothesized that this association in DM patients is mediated by advanced glycation end products (AGEs). Prolonged hyperglycemia in DM patients often results in an increase in oxidation products, such as AGEs, which then mediate increased collagen cross-linking [116]. This subsequently decreases the elasticity of the corneal stroma and potentially prevents corneal thinning [117]. The evidence for this protective effect is, however, mixed. An early case–control study comparing keratoconus patients with normal controls found a significantly lower rate of Type 2 DM in the keratoconus group [118], which was later supported by the findings of several other studies which also determined significant inverse associations between the prevalence of keratoconus and DM [23,27,119]. One study found no significant association between keratoconus and DM but did determine that patients with DM had decreased odds of experiencing progression of their keratoconus to a more severe stage [120]. While these studies suggest a possible protective effect of diabetes against the diagnosis or progression of keratoconus, several other studies have found no association between the two [13,121,122,123] or even higher prevalence of diabetes in the keratoconus population compared to normal controls [24,124].

Recently, several meta-analyses have examined the association between keratoconus and diabetes. One such analysis examined 6 case–control studies and 3 cohort studies and found no significant association between keratoconus and DM, with an odds ratio of 0.87 and a 95% confidence interval of 0.66–1.14 [125]. Another meta-analysis examining 2 additional case–control studies for a total of 8 case–control and 3 cohort studies also determined no significant association between keratoconus and DM, with an odds ratio of 0.83 and 95% confidence interval of 0.66–1.10 [126]. These findings are supported by yet another meta-analysis examining the many risk factors of keratoconus, which also found no significant association between keratoconus and type II DM [127]. Thus, it is still unclear whether DM may confer a protective effect against the development or progression of keratoconus, but it remains an intriguing possibility.

## 4. Summary and Conclusions

In this review of conditions commonly cited to be associated with keratoconus, including atopy, Down syndrome, Ehlers–Danlos syndrome, Marfan syndrome, mitral valve prolapse, osteogenesis imperfecta, and diabetes mellitus, it is evident that the strength of some of these associations is weaker than one might expect based on how often they are cited. For instance, the association between keratoconus and connective tissue disorders has been frequently examined, with the strongest association among connective tissue disorders with keratoconus being mitral valve prolapse. However, the lack of a clear and consistent association between keratoconus and known connective tissue diseases suggests a very different mechanism of pathogenesis from these conditions. The association between keratoconus and Down syndrome is also not entirely clear, with an extremely large variety of prevalence in different populations. Some of this may be due to the lack of sensitive methods of keratoconus detection (i.e., topography or tomography) in early studies, but some of this could be due to an actual absence of association between keratoconus and conditions long thought to be related. Larger case–control studies would help to determine which of the long list of cited associations in Table 1 are likely true, which in turn could help direct new research into the cause of keratoconus.

The strongest evidence of association by far has been found for atopy and keratoconus. Multiple case–control studies have supported this association (Table 2), and additional case–control studies support the association of atopy-related conditions (asthma, allergic rhinitis, eczema, and ocular allergy) with keratoconus (Supplemental Tables S1–S4). While the evidence points towards atopy having the strongest association with keratoconus, there is considerable variation in the data examining specific atopic diseases and their clinical correlation with keratoconus. Still, the strong evidence for a connection between atopy and keratoconus warrants continued investigation into the link between these two conditions and whether treating atopic conditions more aggressively could potentially decrease the chances of developing or worsening keratoconus. Of course, the potentially confounding variable here is eye rubbing. Eye rubbing behavior has also been repeatedly associated with keratoconus. However, the nature of this association is still unclear due to the significant overlap between eye rubbing and ocular allergy, with one condition exacerbating the other. Whether it is the mechanical forces to the cornea from eye rubbing, the release of inflammatory cytokines secondary to eye rubbing that could be contributing to keratoconus progression, or an underlying imbalance of cytokines resulting in altered tissue properties that results in keratoconus irrespective of eye rubbing, or whether keratoconus and its progression occurs primarily and can result in increased ocular inflammation and eye rubbing that has no bearing on the keratoconus, remains to be answered. Even so, advising patients to avoid eye rubbing, as well as reducing ocular surface allergy using lubricants and topical anti-histamines, makes sense due to the low risk of such intervention and the possible benefit. One must, however, be cautious so as not to make patients feel guilty or feel as though they caused their keratoconus by rubbing their eyes, as we cannot be sure that eye rubbing is necessary and sufficient to cause keratoconus.

Finally, there has been interest in the possibility that DM could confer protection against the development of keratoconus, perhaps through promoting endogenous corneal cross-linking. Thus far, the evidence for an inverse relationship between DM and keratoconus is somewhat mixed, but at least four separate studies have found an inverse relationship between DM and keratoconus, and the possibility that having DM could protect against having keratoconus, or perhaps protect against keratoconus progression, is certainly intriguing and warrants continued study.

## Figures and Tables

**Table 1:** Conditions reported to be associated with keratoconus.

Atopic Diseases	
Allergic conjunctivitis	Eczema
Asthma	Hay Fever
**Connective Tissue Diseases**	
Collagen vascular disease	**Marfan syndrome**
**Ehlers–Danlos syndrome**	**Mitral valve prolapse**
Joint hypermobility	**Osteogenesis imperfecta**
**Ocular Diseases**	
Aniridia	Iridoschisis
Ankyloblepharon	Lattice dystrophy
Atopic keratoconjunctivitis	Leber congenital amaurosis
Avellino corneal dystrophy	Leber hereditary optic neuropathy
Axenfeld’s anomaly	Measles retinopathy
Bilateral macular coloboma	Microcornea
Blue sclerae	Ocular hypertension
Brittle corneal syndrome	Pellucid marginal degeneration
Cataracts	Persistent pupillary membrane
Chandler’s syndrome	Posterior lenticonus
Chronic progressive external ophthalmoplegia	Posterior polymorphous dystrophy
Congenital cataracts	Pseudoexfoliative glaucoma
Corneal amyloidosis	Retinitis pigmentosa
Deep filiform corneal dystrophy	Retinopathy of prematurity
Ectopia lentis	Retrolental fibroplasia
Essential iris atrophy	Tapetoretinal degeneration
Fleck corneal dystrophy	Terrien’s marginal degeneration
Floppy eyelid syndrome	Vernal keratoconjunctivitis
**Systemic and Other Disorders**	
Alagille’s syndrome	Mulvihill-Smith syndrome
Albers-Schonberg disease	Nail patella syndrome
Anetoderma	Neurocutaneous angiomatosis
Angelman syndrome	Neurofibromatosis
Apert syndrome	Nonsyndromic autosomal recessive hydrocephalus
Autism spectrum disorders	Noonan syndrome
Autographism	Obesity
Bardet-Biedl syndrome	Obstructive sleep apnea
Congenital hip dysplasia	Oculodentodigital syndrome
Congenital communicating hydrocephalus	Pseudoxanthoma elasticum
Costello syndrome	Raynaud’s syndrome
Crouzon syndrome	Rieger’s syndrome
**Diabetes**	Rheumatoid arthritis
**Down syndrome**	Rothmund’s syndrome
EDICT syndrome	Rubinstein-Taybi syndrome
False chordae tendinae	Scoliosis
Familial Mediterranean Fever	Syndactyly
Focal dermal hypoplasia	Thyroid disease
GAPO syndrome	Tourette syndrome
Goltz-Gorlin syndrome	Turner syndrome
Ichthyosis	Warburg Micro syndrome
Inflammatory bowel disease	Williams-Beuren syndrome
Intellectual disability	Xeroderma pigmentosum
Laurence-Moon-Bardet-Biedl syndrome	X-linked hypohydrotic ectodermal dysplasia
Little’s disease	22q11.2 deletion syndrome
Martsolf syndrome	

Bold font indicates conditions examined in this review.

**Table 2:** Results of case–control studies comparing atopy rates in keratoconus versus control populations.

Study	Population	KCN ^1^ with Atopy	Control with Atopy	Significance
Rahi et al., 1977 [18]	United Kingdom	35% (*n* = 182)	12% (*n* = 100)	Yes (*p* < 0.001)
Wachtmeister et al., 1982 [12]	Sweden	40% (*n* = 30)	23% (*n* = 30)	No (*p* > 0.1)
Bawazeer et al., 2000 [16]	Canada	20% (*n* = 49)	4% (*n* = 71)	Yes (*p* = 0.01)
Millodot et al., 2011 [17]	Israel	40% (*n* = 22)	19% (*n* = 949)	Yes (*p* = 0.01)
Lee et al., 2020 [13]	Korea	20% (*n* = 575)	19% (*n* = 2875)	No (*p* = 0.498)
Ahmed et al., 2021 [15]	Egypt	35% (*n* = 34)	12% (*n* = 66)	Yes (*p* = 0.005)
Meteoukki et al., 2021 [10]	Algeria	69% (*n* = 70)	16% (*n* = 70)	Yes (*p* < 0.001)
Moura et al., 2021 [9]	Brazil	73% (*n* = 30)	11% (*n* = 18)	Yes (*p* < 0.001)
Regueiro et al., 2021 [14]	Spain	60% (*n* = 60)	47% (*n* = 36)	No (*p* = 0.228)
Yang et al., 2022 [19]	China	16% (*n* = 330)	5% (*n* = 330)	Yes (*p* < 0.001)

^1^ KCN = keratoconus.

## Data Availability

No new data were created in the writing of this manuscript.

## Supplementary Materials

The following supporting information can be downloaded at: https://www.mdpi.com/article/10.3390/life13061363/s1, Table S1: Results of case–control studies comparing the rate of asthma in keratoconus and control populations, Table S2: Results of case–control studies comparing the rate of allergic rhinitis in keratoconus and control populations, Table S3: Results of case–control studies comparing rates of eczema and skin allergy in keratoconus and control populations, Table S4: Results of case–control studies comparing the rate of ocular allergy in keratoconus and control populations.
