# Supplementary material for: Systemic Associations with Keratoconus
Source: Life (Basel). 2023 Jun 10;13(6):1363. doi: 10.3390/life13061363 (PMC10304343; doi:10.3390/life13061363)
Supplement: Supplementary file 1 [file life-13-01363-s001.zip › life-2411645-supplementary.pdf]

Supplemental Tables

Table S1. Results of case control studies comparing rate of asthma in keratoconus and control populations.

Article	Population	KCN group size	Control group size	KCN with Asthma	Control with Asthma	Significance
Nemet et al. 2010 [27]	Israel	426	1704	8%	4%	Yes ($p<0.001$)
Woodward et al. 2016 [28]	United States	16053	16053	14%	11%	Yes ($p<0.001$)
Naderan et al. 2017 [26]	Iran	885	1526	5%	1%	Yes ($p<0.001$)
Chang et al. 2020 [21]	Taiwan	3869	23214	4%	4%	Yes $p=0.0316$
Lee et al. 2020 [13]	Korea	575	2875	28%	33%[1]	Yes ($p=0.028$)
Lin et al. 2021 [24]	Taiwan	5055	20220	19%	15%	Yes ($p<0.001$)
Moon et al. 2020 [25]	South Korea	1552	7760	33%	25%	Yes ($p<0.001$)
Claessens et al. 2022 [22]	Netherlands	2051	12306	1.5%	0.6%	Yes ($p<0.001$)
Debourdeau et al. 2022 [23]	France	195	195	20%	8%	Yes ($p<0.001$)

[1]Note that Lee et al. 2020 found a significantly higher rate of asthma in their control population.

Table S2. Results of case control studies comparing rate of allergic rhinitis in keratoconus and control populations.

Article	Population	KCN group size	Control group size	KCN with Allergic Rhinitis	Control with Allergic Rhinitis	Significance
Woodward et al. 2016 [28]	United States	16053	16053	28%	26%	Yes ($p<0.001$)
Naderan et al. 2017 [26]	Iran	885	1526	8%	6%	Yes ($p=0.022$)
Chang et al. 2020 [21]	Taiwan	3869	23214	4%	4%	No ($p=0.4616$)
Lee et al. 2020 [13]	Korea	575	2875	69%	81%[1]	Yes ($p<0.001$)
Lin et al. 2021 [24]	Taiwan	5055	20220	44%	38%	Yes ($p<0.001$)
Moon et al. 2020 [25]	South Korea	1552	7760	78%	64%	Yes ($p<0.001$)
Claessens et al. 2022 [22]	Netherlands	2051	12306	0.4%	0.2%	Yes ($p=0.035$)
Debourdeau et al. 2022 [23]	France	195	195	27%	21%	No ($p=0.12$)

[1]Note that Lee et al. 2020 found a significantly higher rate of allergic rhinitis in their control population.

Table S3. Results of case control studies comparing rate of eczema and skin allergy in keratoconus and control populations.

Article	Population	KCN group size	Control group size	KCN with Eczema/Skin Allergy	Control with Eczema/Skin Allergy	Significance
Naderan et al. 2017 [26]	Iran	885	1526	4%	1%	Yes (p<0.001)
Moon et al. 2020 [25]	South Korea	1552	7760	17%	12%	Yes (p<0.001)

Table S4. Results of case control studies comparing rate of ocular allergy in keratoconus and control populations.

Article	Population	KCN group size	Control group size	KCN with Ocular Allergy	Control with Ocular Allergy	Significance
Naderan et al. 2017[1] [26]	Iran	885	1526	24% (VKC)<br>18% (AC)	4% (VKC)<br>5% (AC)	Yes (p<0.001)
Lee et al. 2020 [13]	Korea	575	2875	36%	31%	Yes (p<0.035)
Debourdeau et al. 2022 [23]	France	195	195	27%	21%	No (p=0.12)

[1]Rates for vernal keratoconjunctivitis (VKC) and allergic conjunctivitis (AC) were reported separately. There were significant differences found for both VKC and AC.
